# Supplementary material for: Utilization of preconception care and associated factors in Hosanna Town, Southern Ethiopia
Source: PLoS One. 2022 Jan 7;17(1):e0261895. doi: 10.1371/journal.pone.0261895 (PMC8741054; doi:10.1371/journal.pone.0261895)
Supplement: S2 File — (DOCX) [file pone.0261895.s002.docx]

English Version Questionnaire

Hello! I am MPH/Epidemiology student at Woliata Sodo University, College of Medicine & Health Sciences, School of Public Health, and doing research on preconception care utilization and associated factors among pregnant women attending antenatal care clinics of public health facilities in Hosanna town. The aim of the study is to assess preconception care utilization and associated factors among pregnant women attending antenatal care clinics of public health facilities in Hosanna town, 2020. If you agree to participate in this study, you will be required to answer a series of question that have been prepared for the study through interview in order to obtain the intended information. The interview will take approximately 25-30 minutes. All information that will be collected will be kept in private and will be used only for this study. The form will not bear your name but identification number. Participating in this study is completely voluntary. You have a right not to participate in this study and even if you have already accept to participated in the study you can quit at any time if you feel so. Refusal to participate or withdrawal from the study will not involve penalty or loss of any benefits.

If you agree to participate in the study, please sign below.

Signature______________________

If, no respect the decision and thank her. If yes continue the interview.

**Result**

Completed----------------------

Respondent not available-------------------

Refused------------------------------

Partially completed---------------------

Other (please specify) -------------------

Name of the interviewer ……………………. Signature………. Date……………..

Name of the supervisor ……………………. Signature………. Date……………..

Annex 3: Questionnaire English Version

**English version questionnaire for the study on preconception care utilization and associated factors among pregnant women (quantitative part).**

Date of interview ___/__/ 2020Name of the facility.................... Questionnaire code.........

**SECTION 1: Socio – demographic information**

| **S.no** | \| **Demographic Questions** \| \| --- \| | **Responses** | **Skip** | **Code** |
| --- | --- | --- | --- | --- | --- |
|  | How old are you? (In completed years) | ------------------------------ |  |  |
|  | What is your marital status? | - 1. Married - 2. Single - 3. Divorced - 4. Widowed |  |  |
|  | What is your ethnicity? | - 1. Hadiya - 2. Kembata - 3. Silte - 4. Gurage - 5. Amhara - 6. Oromo - 7. Others (specify)……... |  |  |
|  | What is your religion? | - 1. Orthodox - 2. Muslim - 3. Protestant - 4. Catholic - 5. Others (specify)……........ |  |  |
|  | What is your level of educational status? | - 1. No formal education - 2. Read and write - 3. Primary education - 4. Secondary education - 5. Diploma and more |  |  |
|  | What is your husband’s level of educational status? | - 1. No formal education - 2. Read and write - 3. Primary education - 4. Secondary education - 5. Diploma and more |  |  |
|  | What is your Occupation? | - 1. Government employee - 2. Merchant - 3. Farmer - 4. House wife - 5. Daily laborer - 6. Others (specify)……....... |  |  |
|  | What is your husband’s occupation? | - 1. Government employee - 2. Merchant - 3. Farmer - 4. Daily laborer - 5. Others (specify)……....... |  |  |
|  | How much is your monthly income? | --------------------------- Birr |  |  |
|  | Where is your place of residence? | - 1. Urban - 2. Rural |  |  |

**SECTION 2: Reproductive & Medical Characteristics**

| **S.no** | \| **Questions** \| \| --- \| | **Responses** | **Skip** | **Code** |
| --- | --- | --- | --- | --- | --- |
|  | How many times have you been pregnant so far including this pregnancy? (Gravidity) | ----------------------------- |  |  |
|  | Have you ever given birth? | - 1. Yes - 2. No | If No, go to Q 22 |  |
|  | How many times have you given birth whether it’s alive or dead? (Parity) | ----------------------------- |  |  |
|  | How many were alive at birth? | ----------------------------- |  |  |
|  | How many were dead at birth? | ----------------------------- |  |  |
|  | Have you ever given births who were born alive but later died? | - 1. Yes - 2. No | If No, go to Q 18 |  |
|  | How many children died later after born alive? | ----------------------------- |  |  |
|  | How many living children do you have? | ----------------------------- |  |  |
|  | How old are you when you gave birth to your first child? (In completed years) | ----------------------------- |  |  |
|  | When did you give birth to your last child? | ----/-------/----------- |  |  |
|  | When did you give birth to your child before the last child? | ----/-------/----------- |  |  |
|  | How many months are you pregnant now? | -------------------------------- |  |  |
|  | How many times did you receive antenatal care during this pregnancy? | ----------------------------- |  |  |
|  | Have you ever used family planning? | - 1. Yes - 2. No |  |  |
|  | Which method have you used?(multiple responses are possible) | - 1. Female sterilization - 2. Male sterilization - 3. IUCD - 4. Injectable - 5. Implants - 6. Pill - 7. Male condom - 8. Female condom - 9. Emergency contraception - 10. Calendar method - 11. Lactation Amenorrhea Method - 12. Withdrawal |  |  |
|  | Have you used family planning before current pregnancy? | - 1. Yes - 2. No | If No, go to Q 28 |  |
|  | Which method have you used? | - 1. Female sterilization - 2. Male sterilization - 3. IUCD - 4. Injectable - 5. Implants - 6. Pill - 7. Male condom - 8. Female condom - 9. Emergency contraception - 10. Calendar method - 11. Lactation Amenorrhea Method - 12. Withdrawal |  |  |
|  | Have you ever experienced complications during pregnancy | - 1. Yes - 2. No | If Primigravidas, go to Q.32 |  |
|  | Which type of complication during pregnancy have you experienced? (multiple responses are possible) | - 1. Antepartum hemorrhage - 2. Preeclampsia - 3. Eclampsia - 3. Gestational DM - 4. Postpartum hemorrhage - 5.RH incompatibility - 6. Complications during labour & delivery - 7. Intrauterine growth restriction - 8. C/S delivery - 9. Others (specify)………... |  |  |
|  | Have you ever experienced adverse pregnancy outcomes? | - 1. Yes - 2. No | If No, go to Q.32 |  |
|  | Which type of adverse pregnancy outcome have you experienced? (multiple responses are possible) | - 1. Congenital anomalies - 2. Low birth weight - 3. Preterm - 4. Abortion - 5. Still birth - 6. Neonatal death - 7. Others (specify)……....... |  |  |
|  | Do have medically confirmed diseases? | - 1. Yes - 2. No | If No, go to Q.35 |  |
|  | If yes, which type of medical disease do you have? (multiple responses are possible) | - 1. Diabetes mellitus - 2. Chronic hypertension - 3. Chronic renal disease - 4. Asthma - 5. Cardiac disease - 4. HIV/AIDS - 5. Others (specify)……....... |  |  |
|  | Do you have family history of medically confirmed diseases? | - 1. Yes - 2. No | If No, go to next section |  |
|  | If yes, which type of medical disease do your families have? (multiple responses are possible) | - 1. Diabetes mellitus - 2. Chronic hypertension - 3. Chronic renal disease - 4. Asthma - 5. Cardiac disease - 6. Others (specify)……....... |  |  |
|  | Have you ever planned for any of your previous pregnancies? | - 1. Yes - 2. No |  |  |
|  | Is your current pregnancy planned? | - 1. Yes - 2. No |  |  |

**SECTION 3: Health service related factors on**

| **S.no** | \| **Questions** \| \| --- \| | **Responses** | **Skip** | **Code** |
| --- | --- | --- | --- | --- | --- |
|  | How much is the distance between your home & this facility? (in KMs) | ………………………………. |  |  |
|  | Do you have a challenge in accessing this health facility? | - 1. Yes - 2. No |  |  |
|  | Have you received counseling on life style modification from a facility previously? | - 1. Yes - 2. No |  |  |
|  | Which type of counseling have you received? | - 1. Counseling on folic acid supplementation - 2. Counseling to quit smoking, alcohol before conception - 3. Weight management before conception - 4. Diet modification before conception - Others (specify)……....... |  |  |

**SECTION 4: Knowledge of mothers on preconception care**

| **S.no** | \| **Knowledge Questions** \| \| --- \| | **Response** | **Skip** | **Code** |
| --- | --- | --- | --- | --- | --- |
|  | Women’s health and lifestyle before pregnancy can influence both the fertility and the health of mother and child. | - 1. Yes - 2. No - 3. I don’t know |  |  |
|  | Do you know that women should prepare & maintain their health before getting pregnant | - 1. Yes - 2. No - 3. I don’t know |  |  |
|  | Have you ever heard about preconception care? | - 1. Yes - 2. No | If no go to Q. 46 |  |
|  | From where did you get the information?(multiple responses are possible) | - 1. Family members/Relatives - 2. Friends - 3. Health professionals including HEW - 4. Traditional birth attendants - 5. Women development army - 6. Mass Media (Radio, TV, newspaper, social media) - 7. Neighbors - 8. Others (specify) ………. |  |  |
|  | Do you know things that should be done before pregnancy? | - 1. Yes - 2. No | If no go to Q. 48 |  |
|  | If yes, what sort of things should women/couples do before pregnancy? (multiple responses are possible) | - 1. Planning pregnancy - 2. Visiting health facility to seek advice on healthy pregnancy - 3. Being screened & treated for chronic medical disease - 4. Being screened & treated for STIs including HIV - 5. Being diagnosed & treated for infertility - 6. Getting vaccination for tetanus - 7. Using family planning for spacing children - 8. Taking folic acid - 9. Weight management - 10. Diet modification - 11. Quit smoking & drinking alcohol - 12. Avoiding teratogenic & illicit drugs - 13.Protection from unnecessary chemical/radiation exposure in occupational, environmental and medical settings - 14. Genetic counseling, screening & treatment of genetic conditions when appropriate - 15. Providing health care services, referral and psychosocial support to victims of violence - 16. Counseling on controlling preexisting chronic medical condition before getting pregnant - 17. Counseling to get treatment for previous adverse pregnancy & birth outcomes before getting pregnant - 16. Others (specify)……………… |  |  |
|  | Which chronic medical conditions affect the fetus? (multiple responses are possible) | - 1. Diabetes mellitus - 2. Chronic hypertension - 3. Chronic renal disease - 4. Asthma - 5. Cardiac disease - 6. STIs including HIV - 7. Genetic disease - 7. Epilepsy - 8. Depression - 9. I don’t know |  |  |
|  | Which life style or behavioral or environmental conditions affect the fetus? (multiple responses are possible) | - 1. Smoking cigarette - 2. Drinking alcohol - 3. Taking illicit drugs - 4. Being over-weight/under-weight - 5. Exposure to radiation or chemicals - 6. Being victim of gender based violence - 7. I don’t know |  |  |
|  | For whom do you think preconception care and counseling is needed? | - 1. For teenagers - 2. For reproductive age women - 3. For women with chronic medical illness - 4. For women with previous pregnancy complication or adverse birth outcomes - 5. For couples - 6. For married couples - 7. For all women - 8. I don’t know |  |  |
|  | Do you know the benefit of preconception care services? | - 1. Yes - 2. No |  |  |
|  | If yes for Q.51, what are its benefits? | - 1. Improve maternal health - 2. Improve pregnancy outcome - 3. Reduced unplanned pregnancy - 4. I don’t know |  |  |
|  | For whom preconception care is important? | - 1. For baby, only - 2. For mother, only - 3. For baby and mother - 4. Don’t know |  |  |
|  | When do women/couples should receive preconception care? | - 1. When planning to get pregnant - 2. 3 months before being pregnant - 3. During the first 3 months of pregnancy - 4. 3 months before being pregnant - 5. During the first 3 months of pregnancy - 6. Between pregnancies - 7. I don’t know |  |  |
|  | Who is the appropriate person for providing preconception care? | - 1. Doctor - 2. Health Officer - 3. Midwife - 4. Health extension worker - 5. All are appropriate - 6. I don’t know |  |  |
|  | Do you know where preconception care services are provided? | - 1. Public hospitals - 2. Health centers - 3. Health post - 4. Private clinic - 5. Home - 6. I don’t know |  |  |
|  | How frequently need these services to be provided? | - 1. Sometimes - 2. Usually - 3. Continuously - 4. I don’t know |  |  |
|  | Do you know when preconception care services are provided in general? | - 1. Immediately before pregnancy - 2. During first 2 months of pregnancy - 3. During delivery - 4. Immediate post-partum - 5. Between pregnancies - 6. When the women/couples ready to conceive - 7. At any time couples plan to get pregnant - 8. I don’t know |  |  |

**Section 5: Attitude of mothers on preconception care**

| **S. no** | \| **Attitude Questions** \| \| --- \| | **Responses** | | | | |
| --- | --- | --- | --- | --- | --- | --- | --- |
|  |  | **SA** | **A** | **N** | **D** | **SD** |
|  | Preconception care is a high health care priority for all women/couples planning pregnancy. |  |  |  |  |  |
|  | Women with medically confirmed diseases should only receive preconception care services. |  |  |  |  |  |
|  | Women who had pregnancy complications or adverse birth outcomes previously should only receive preconception care services. |  |  |  |  |  |
|  | Preconception care services should be provided for reproductive age women |  |  |  |  |  |
|  | Preconception care services should be provided for married women |  |  |  |  |  |
|  | Preconception care services should be provided for unmarried women |  |  |  |  |  |
|  | Preconception care services are not important for adolescent girls |  |  |  |  |  |
|  | Husbands should accompany their wives while seeking any preconception care services. |  |  |  |  |  |
|  | Preconception care services should be provided by male health professionals. |  |  |  |  |  |
|  | Preconception care services should be provided by female health professionals. |  |  |  |  |  |
|  | Preconception care services should be provided by traditional birth attendants |  |  |  |  |  |
|  | Preconception care does not have any effect on birth outcome. |  |  |  |  |  |

**Section 6: Utilization of preconception care**

| **S.no** | \| **Utilization Questions** \| \| --- \| | **Responses** | **Skip** | **Code** |
| --- | --- | --- | --- | --- | --- |
|  | Have you ever received preconception care service from health facility during any of your previous pregnancy? | - 1. Yes - 2. No | If No, go to Q.73 |  |
|  | If yes Q. 71, which preconception care services have you received? | - 1. Being screened & treated for chronic medical diseases - 2. Being screened & treated for STIs - 3. HIV counseling, testing & initiating HAART if reactive - 4. Being diagnosed & treated for infertility/sub-fertility - 5. Getting vaccination for tetanus - 6. Taking folic acid supplementation - 7. Had follow up and care for preexisting chronic medical condition before getting pregnant - 8. Had follow up and care for previous adverse pregnancy & birth outcomes before getting pregnant - 9. Weight management - 10. Diet modification - 11. Avoiding smoking & drinking alcohol - 12. Avoiding teratogenic & illicit drugs - 13.Avoiding chemical/radiation exposure in occupational, environmental and medical settings |  |  |
|  | Have you received preconception care service from health facility in the current pregnancy? | - 1. Yes - 2. No |  |  |
|  | If yes to Q.73, which preconception care services had you received in the current pregnancy? | - 1. Being screened & treated for chronic medical diseases - 2. Being screened & treated for STIs - 3. HIV counseling, testing & initiating HAART if reactive - 4. Being diagnosed & treated for infertility/sub-fertility - 5. Getting vaccination for tetanus - 6. Taking folic acid supplementation - 7. Had follow up and care for preexisting chronic medical condition before getting pregnant - 8. Had follow up and care for previous adverse pregnancy & birth outcomes before getting pregnant - 9. Weight management - 10. Diet modification - 11. Avoiding smoking & drinking alcohol - 12. Avoiding teratogenic & illicit drugs - 13.Avoiding chemical/radiation exposure in occupational, environmental and medical settings |  |  |
|  | When did you receive preconception care service in the current pregnancy? | - 1. When I planned to get pregnant - 2. 3 months before being pregnant - 3. During the first 3 months of pregnancy - 4. Between pregnancies - 5. Others (specify)………… |  |  |
|  | In which unit have you received the preconception care service in the current pregnancy? | - 1. Family planning unit - 2. Post natal care unit - 2. Gyne-OPD - 3. Chronic disease follow-up clinic - 4. Others (specify)……… |  |  |
|  | Where did you receive the preconception care service in the current pregnancy? | - 1. Public hospital - 2. Health center - 3. Health post - 4. Private clinic - 5. Others (specify)……… |  |  |
|  | Who provided you the preconception care service in the current pregnancy? | - 1. Doctor - 2. Nurse - 3. Midwife - 4. Health officer - 5. Health extension worker - 6. Others (specify)……… |  |  |
|  | Who makes decision on pregnancy planning & seeking health care services? | - 1. Women alone - 2. Husband alone - 3. Women & Husband - 4. Others (specify………….) |  |  |
|  | Did your husband supports & accompany you while having preconception care services? | - 1. Yes - 2. No |  |  |
|  | How many times did you receive preconception care services in the current pregnancy? | -------------------------------------------- |  |  |
|  | If you were screened for chronic medical conditions as preconception care services in the current pregnancy, which condition had you screened? | - 1. Hypertension - 2. Diabetes - 3. Tuberculosis - 4. Anemia - 5. Epilepsy - 6. Infertility - 7. Asthma - 8. Others |  |  |
|  | If you managed your weight as preconception care services in the current pregnancy, what kind of weight management have you done? | - 1. Maintained healthy weight - 2. Lost weight |  |  |
|  | If you took folic acid supplementation, have you consistently used them? | - 1. Yes - 2. No |  |  |
|  | If you modify your diet as preconception care services in the current pregnancy, what kind of modifications have you made? | - 1. Foods with high-carbohydrate avoided - 2. Foods with high saturated fat avoided - 3. Foods with high glycemic avoided - 4. Eating balanced diet& increased meal frequency with diversified food items - 5. Others (specify-------------) |  |  |
