# Supplementary material for: Utilization of preconception care and associated factors in Hosanna Town, Southern Ethiopia
Source: PLoS One. 2022 Jan 7;17(1):e0261895. doi: 10.1371/journal.pone.0261895 (PMC8741054; doi:10.1371/journal.pone.0261895)
Supplement: S3 File — (DOCX) [file pone.0261895.s003.docx]

**ፍቃድ መጠየቂያ ቅፅ**

ጤና ይስጥልኝ እኔ በወላይታ ሶዶ ዩኒቨርሲቲ ህክምክና ጤና ሳይንስ ኮሌጅ ህብረተሰብ ጤና አጠባበቅ ትምህርት ቤት የማሰተርስ ተማሪ ነኝ፡፡ በአሁኑ ሰአት በሆሳዕና ከተማ በሚገኙ የመንግስት የጤና ተቋማት ዉስጥ ባሉ ነፍስ ጡር እናቶች ላይ ከእርግዝና በፊት በሚደረግ የጤና ክትትል ዙሪያ ጥናት እያደርኩ እገኛለሁ፡፡ የጥናቱ ዓላማ በሆሳዕና ከተማ በሚገኙ የመንግስት የጤና ተቋማት ዉስጥ ያሉ ነፍሰ ጡር እናቶች ከእርግዝና በፊት በሚደረግ የጤና ክትትል ዙሪያ ያላቸዉ አጠቃቀም እና ተዛማጅ ነገሮች መዳሰስ ነዉ፡፡ እዚህ ጥናት ላይ ለመሳተፍ ከተስማሙ የሚፈልገዉን መረጃ ለማግኘት ሲባል ተከታታይ ጥያቄዎች መልስ መስጠት ይጠበቅብዎታል፡፡ ይህ ቃለመጠይቅ ከ 20-25 ደቂቃ ሊወስድ ይችላል ፡፡ የሚሰበሰቡትም መረጃዎች በሙሉ በሚስጢር የሚቀመጡ ሲሆን ለዚህ ጥናት ብቻ ያገለግላሉ፡፡  ቅጹ የመለያ ኮድ እንጂ ስምዎን አይይዝም፡፡  በዚህ ጥናት ላይ የሚሳተፉት ሙሉ በሙሉ ፈቃደኛ ከሆኑ ብቻ ነው፡፡ በእዚህ ጥናት ያለመሳተፍ መብት አልዎት እናም በጥናቱ ላይ ለመሳተፍ ቀድሞውኑ የተቀበሉ ቢሆኑም በመሃል ማቐርጥ የሚፈልጉ ከሆነ በማንኛውም ጊዜ ማቆም ይችላሉ፡፡ በጥናቱ አለመሳተፍ ወይም መሃል ማቋረጥ ማንኛውንም ጥቅሞችን አያስቀርም ወይም ቅጣትን አያስከትልም፡፡

በጥናቱ ለመሳተፍ ከተስማሙ እባክዎን ከዚህ በታች ይፈርሙ፡፡

ፊርማ______________________

ፍቃደኛ ካልሆኑ ውሳኔውን አክብረህ አሰናብት፡፡ አዎ ከሆነ ቃለ-መጠይቁን ይቀጥሉ።

**ውጤት**

ተጠናቅቋል ----------------------

መልስ ሰጪ አልተገኘም -------------------

ውድቅ ተደርጓል ------------------------------

በከፊል ተጠናቅቋል ---------------------

ሌላ (እባክዎን ይግለጹ) -------------------

የቃለ-መጠይቁ የጠየቀዉ ስም ……………………. ፊርማ ………. ….ቀን …………… ..

የተቆጣጣሪው ስም ……………………. ፊርማ ……………………. ቀን …………… ..

**Annex 5: Questionnaire Amharic Version**

**በሆሳዕና ከተማ በሚገኙ የጤና ተቐማት ዉስጥ ያሉ ነፍሰ ጡር እናቶች ከእርግዝና በፊት በሚደረግ የጤና ክትትል ዙሪያ ያላቸዉ አጠቃቀም እና ተዛማጅ ነገሮች**

**የቃለ-መጠይቁ ቀን ___ / __ / 2012 የጤና ተቋሙ ስም ……………… የቃለ-መጠይቁ ኮድ.........**

**ክፍል 1 ፡-ግለ-ታሪክ መረጃን የሚዳስሱ ጥያቄዎች**

| **ተ.ቁ** | \| **ጥያቄ** \| \| --- \| | **ምላሾች** | **ዝለል** | **ኮድ** |
| --- | --- | --- | --- | --- | --- |
|  | ዕድሜዎ ስንት ነው? (በተጠናቀቁ ዓመታት) | ------------------------------ |  |  |
|  | የጋብቻ ሁኔታዎ ምንድን ነው? | - 1. ያገባች - 2. ያላገባች - 3. የፈታች - 4. ባሏ የሞተ |  |  |
|  | ብሄሮ ምንድን ነው? | - 1. ሀዲያ - 2. ካምባታ - 3. ስልጤ - 4. ጉራጌ - 5. አማራ - 6. ኦሮሞ - 7. ሌሎች (ይግለጹ) ……… |  |  |
|  | ሃይማኖትዎ ምንድነው? | - 1. ኦርቶዶክስ - 2. ሙስሊም - 3. ፕሮቴስታንት - 4. ካቶሊክ - 5. ሌሎች (ይግለጹ) ……...... |  |  |
|  | የትምህርት ደረጃዎ ምን ያህል ነው? | - 1. መደበኛ ትምህርት ያልወሰደ - 2. ማንበብ እና መጻሀፍ የሚችል - 3. የመጀመሪያ ደረጃ ትምህርት ያጠናቀቀ - 4. ሁለተኛ ደረጃ ትምህርት ያጠናቀቀ - 5. ዲፕሎማ እና ከዛ በላይ |  |  |
|  | የባልሽ የትምህርት ደረጃ ምን ያህል ነው? | - 1. መደበኛ ትምህርት ያልወሰደ - 2. ማንበብ እና መጻሀፍ የሚችል - 3. የመጀመሪያ ደረጃ ትምህርት ያጠናቀቀ - 4. ሁለተኛ ደረጃ ትምህርት ያጠናቀቀ - 5. ዲፕሎማ እና ከዛ በላይ |  |  |
|  | የሥራ መስክዎ ምንድን ነው | - 1. የመንግስት ሰራተኛ - 2. ነጋዴ - 3. ገበሬ - 4. የቤት እመቤት - 5. ዕለታዊ ሰራተኛ - 6. ሌሎች (ይግለጹ) ……....... |  |  |
|  | የባልሽ ሥራ ምንድን ነው? | - 1. የመንግስት ሰራተኛ - 2. ነጋዴ - 3. ገበሬ - 4. ዕለታዊ ሰራተኛ - 5. ሌሎች (ይግለጹ) ……....... |  |  |
|  | ወርሃዊ ገቢዎ ስንት ነው? | --------------------------- ብር |  |  |
|  | የመኖሪያ ቦታዎ የት ነው? | - 1. ከተማ - 2. ገጠር |  |  |

**ክፍል 2:-የስነ-ተዋልዶ እና የጤና እክልን የሚዳስሱ ጥያቄዎች**

| **S.no** | \| **Questions** \| \| --- \| | **Responses** | **Skip** | **Code** |
| --- | --- | --- | --- | --- | --- |
|  | ይህንን እርግዝና ጨምሮ እስከ አሁን ምን ያህል ጊዜ ነፍሰ ጡር ሆነዋል? | ----------------------------- |  |  |
|  | ልጅ ወልደዉ ያዉቃሉ? | - 1. አዎ - 2. አላዉቅም | መልሶ አላዉቅም ከሆነ ወደ ጥ.ቁ. 22 ይሂዱ |  |
|  | ቡህይወት ያለ ልጅ ይሁን የሞተ ስንት ጊዜ ነው የወለዱት? | ----------------------------- |  |  |
|  | በሚወለዱበት ጊዜ በሕይወት የነበሩት ስንት ነበሩ? | ----------------------------- |  |  |
|  | በሚወለዱበት ጊዜ ስንት ህጻንት ሞተዋል? | ----------------------------- |  |  |
|  | በሕይወት የተወለዱ በኋላ ግን የሞቱ ልጆች አሎዎት? | - 1. አዎ - 2. የለኝም | መልሶ የለኝም ከሆነ ወደ ጥ.ቁ. 19 ይሂዱ |  |
|  | በህይወት ከተወለዱ በኋላ ስንት ልጆች ሞቱ? | ----------------------------- |  |  |
|  | በአሁኑ ሰዓት ስንት በሕይወት ያሉ ልጆች ስንት ናቸዉ? | ----------------------------- |  |  |
|  | የመጀመሪያ ልጅዎን ሲወለዱ ስንት ዓመቶት ነበር? | ----------------------------- |  |  |
|  | የመጨረሻ ልጅዎን መቼ ነበር የወለዱት? | ----/-------/----------- |  |  |
|  | ከመጨረሻው ልጆ በፊት ያለዉን ልጅዎን መቼ ነበር የወለዱት ? | ----/-------/----------- |  |  |
|  | አሁን ስንት ወራት እርጉዝ ነዎት? | -------------------------------- |  |  |
|  | በዚህ እርግዝና ወቅት ስንት ጊዜ የእርግዝና ክትትል አግኝተዋል? | ----------------------------- |  |  |
|  | የቤተሰብ ምጣኔ ተጠቅመዉ ያዉቃሉ? | - 1. አዎ - 2. አላዉቅም |  |  |
|  | የትኛውን ዘዴ ነው የተጠቀሙት? (ብዙ ምላሾች ሊኖሩ ይችላሉ) | - 1. የሴት ማህጸን ቱቦ ማስቋጠር - 2. የወንድ ዘር ማስተላለፊያ ቱቦ ማስቋጠር - 3. ማህጸን ዉስጥ የሚቀበር ሉፕ - 4. መርፌ - 5. በቆዳ ስር የሚቀበር የእርግዝና መከላከያ - 6. የእርግዝና መከላከያ እንክብል - 7. የወንድ ኮንደም - 8. የሴት ኮንደም - 9. ድንገተኛ የእርግዝና መከላከያ - 10. የካሌንደር ዘዴ - 11. ጡት የማጥባት የእርግዝና መከላከያ ዘዴ - 12. በግኑኘነት ወቅት የወንድ ዘር ፈሳሽ ወደ ዉጭ ማፍሰስ |  |  |
|  | አሁን ካለው እርግዝና በፊት የቤተሰብ ምጠኔ ተጠቅመዋል? | - 1. አዎ - 2. አልተጠቀምኩም |  |  |
|  | መልሶ አዎን ከሆነ የትኛው ዘዴ ተጠቅመዋል? (ብዙ ምላሾች ሊኖሩ ይችላሉ) | - 1. የሴት ማህጸን ቱቦ ማስቐጠር - 2. የወንድ ዘር ማስተላለፊያ ቱቦ ማስቐጠር - 3. ማህጸን ዉስጥ የሚቀበር ሉፕ - 4. መርፌ - 5. በቆዳ ስር የሚቀበር የእርግዝና መከላከያ - 6. የእርግዝና መከላከያ እንክብል - 7. የወንድ ኮንደም - 8. የሴት ኮንደም - 9. ድንገተኛ የእርግዝና መከላከያ - 10. የካሌንደር ዘዴ - 11. ጡት የማጥባት የእርግዝና መከላከያ - 12. በግኑኘነት ወቅት የወንድ ዘር ፈሳሽ ወደ ዉጭ ማፍሰስ |  |  |
|  | በእርግዝና ወቅት ክእርግዝና ጋር የተያያዙ ችግሮች አጋጥመው ያውቃሉ? | - 1.አዎ - 2. አያዉቅም | ለመጀመሪያ ጊዜ ከሆነ ያረገዘችዉ ወደ ጥ.ቁ. 32 ይሂዱ |  |
|  | መልሶ አዎን ከሆነ በእርግዝና ወቅት ክእርግዝና ጋር የተያያዙ ምን አይነት ችግሮች ገጥሞዎታል? (ብዙ ምላሾች ሊኖሩ ይችላሉ) | - 1. የቅድመ ወሊድ ደም መፍሰስ - 2. ከእርግዝና የተያያዘ የደም ግፊት መጨመር - 3. ከእርግዝና የተያያዘ የደም ግፊት መጨመር እና ራስን ስቶ መዉደቅ - 4. ከእርግዝና የተያያዘ የስኳር ህመም - 5. የድህረ ወሊድ ደም መፍሰስ - 5.ሾተላይ - 6. በምጥና በወሊድ ጊዜ የሚከሰት ችግር - 7. በማኅፀኑ ዉስጥ የሚከስት የጽንስ መቀጨጭ - 8. በኦፕሬሽን መዉለድ - 9. ልላ (ይገለጽ)………... |  |  |
|  | ከዚህ በፊት ሲወለዱ ከህጻኑ ጋር ተያይዞ እክል አጋጥሞውት ያውቃል? | - 1. አዎ - 2. አያዉቅም | መልሶ አያዉቅም ከሆነ ወደ ጥ.ቁ. 33 ይሂዱ |  |
|  | መልሶ አዎን ከሆነ የትኘዉ አያነት ከህጻን ጋር የተያያዘ እክል ገጥሞዎት ነበር? (ብዙ ምላሾች ሊኖሩ ይችላሉ) | - 1. የአፈጣጠር ችግር ያለበት ህጻን - 2. ዝቅተኛ ክብደት ያለዉ ህጻን - 3. ግዜዉ ሳይደርስ የተወለደ ህጻን - 4. ፅንስ ማስወረድ - 5. ሞቶ የተወለደ ህጻን - 6. የጨቅላ ህጻን ሞት |  |  |
|  | በሕክምና የተረጋገጠ በሽታ አለቦዎት? | - 1. አዎ - 2. የለብኝም | መልሶ የለብኝም ከሆነ ወደ ጥ.ቁ. 35 ይሂዱ |  |
|  | አዎ ከሆነ ፣ የትኛው ዓይነት በሽታ አለብዎት? (ብዙ ምላሾች ሊኖሩ ይችላሉ) | - 1. የስኳር ህመም - 2. ሥር የሰደደ የደም ግፊት - 3. ሥር የሰደደ የኩላሊት በሽታ - 4. አስም - 5. የልብ በሽታ - 4. ኤች አይ ቪ / ኤድስ - 5. ሌላ (ይግለጽ)……....... |  |  |
|  | ከቤተሰቦ በሕክምና የተረጋገጠ በሽታ ያለበት ሰዉ አለ? | - 1. አዎ - 2. የለም | መልሶ የለም ከሆነ ወደ ሚቀጥለዉ ክፍል ይሂዱ |  |
|  | አዎ ከሆነ ፣ የትኛው ዓይነት በሽታነዉ ያለበት? (ብዙ ምላሾች ሊኖሩ ይችላሉ) | - 1. የስኳር ህመም - 2. ሥር የሰደደ የደም ግፊት - 3. ሥር የሰደደ የኩላሊት በሽታ - 4. አስም - 5. የልብ በሽታ - 6. ኤች አይ ቪ / ኤድስ - 7. ሌላ (ይግለጽ)……....... |  |  |
|  | ከዚህ በፊት ከነበሩት እርግዝናዎች መካከል አቅደዉ ያረገዙት ነበር? | - 1. አዎ - 2. አልነበረም |  |  |
|  | የአሁኑ እርግዝና አቅደዉ ነዉ ያረገዙት ወይ? | - 1. አዎ - 2. አይደለም |  |  |

**ክፍል 3፡ የጤና አገልግሎት ነክ ጉዳዮች የተመለከቱ ጥያቄዎች**

| **ተ.ቁ** | \| **ጥያቄ** \| \| --- \| | **ምላሾች** | **ዝለል** | **ኮድ** |
| --- | --- | --- | --- | --- | --- |
|  | በቤትዎ እና በዚህ ተቋም መካከል ያለው ርቀት ምን ያህል ነው? (በ ኪ.መ) | ………………………………. |  |  |
|  | ወደዚህ የጤና ተቋም ለመምጣት ከተደራሽነት አኳያ የገጠሞት ፈታና አለ ወይ? | - 1. አዎ - 2. የለም |  |  |
|  | ከዚህ በፊት ከጤና ተቋም የአኗኗር ዘይቤ ማሻሻልን በተመለከተ ምክር አግኝተዋልን? | - 1. አዎ - 2. አልተቀበልኩም | መልሶ አልተቀበልኩም ከሆነ ወደ ሚቀጥለዉ ክፍል ይሂዱ |  |
|  | ምን ዓይነት የምክር አገልግሎት አግኝተዉ ነበር? | - 1. ከመፀነስ በፊት/በኋላ ፎሊክ አሲድ የሚባል መዳሓኒት መወሰድ እንዳለበት ተመክሬ ነበር - 2. ከመፀነስ በፊት/በኋላ ሲጋራ ማጨስ ፣ አልኮል መጠጣት መቆም እንዳለበት ተመክሬ ነበር - 3. ከመፀነስ በፊት የሰዉነት ክብደትን ማሰተካከል እንደሚገባ ተመክሬ ነበር - 4. ከመፀነስ በፊት/ በኋላ አመጋገብ ሁኔታን ማሻሻል እንደሚገባ ተመክሬ ነበር - 5. ሌላ (ይግለጽ)……....... |  |  |

**ክፍል 4፡ በቅድመ ፅንስ እንክብካቤ ላይ የእናቶች እውቀት የተመለከቱ ጥያቄዎች**

| **ተ.ቁ** | \| **ጥያቄ** \| \| --- \| | **ምላሾች** | **ዝለል** | **ኮድ** |
| --- | --- | --- | --- | --- | --- |
|  | ከእርግዝና በፊት የሴቶች ጤና እና አኗኗር ሁኔታ በእናቶች እና የልጆች ጤና ላይም ሆነ በመጸነስ ላይ ተጽዕኖ ሊያሳድር ይችላል | - 1. አዎ - 2. አይችልም - 3. አላውቅም |  |  |
|  | ሴቶች ከመፀነሳቸዉ በፊት ጤናቸውን ማዘጋጀት እና መጠበቅ እንዳለባቸው ያውቃሉ? | - 1. አዎ - 2. የለባቸዉም - 3. አላውቅም |  |  |
|  | ስለ ቅድመ ፅንስ እንክብካቤ ሰምተው ያውቃሉ? | - 1. አዎ - 2. አላውቅም | መልሶ አላውቅም ከሆነ ወደ ጥ.ቁ. 46 ይሂዱ |  |
|  | መረጃውን ከየት አገኙት? (ብዙ ምላሾች ሊኖሩ ይችላሉ) | - 1. የቤተሰብ አባላት / ዘመዶች - 2. ጓደኞች - 3. ከጤና ባለሙያዎች የጤና ኤክስቴሽን ባለሙያዎች ጨምሮ - 4. ከልምድ አዋላጆች - 5. ከሴቶች የልማት ሰራዊት - 6. ከመገናኛ ብዙሃን (ሬዲዮ ፣ ቴሌቪዥን ፣ ጋዜጣ ፣ ማህበራዊ ሚዲያ) - 7. ከጎረቤቶች - 8. ሌሎች (ይግለጹ) ………. |  |  |
|  | ከእርግዝና በፊት ምን መደረግ እንዳለባት ያውቃሉ? | - 1. አዎ - 2. አላውቅም | መልሶ አላውቅም ከሆነ ወደ ጥ.ቁ. 48 ይሂዱ |  |
|  | አዎ ከሆነ ፣ ሴቶች / ባለትዳሮች ከእርግዝና በፊት ምን ዓይነት ነገሮችን ማድረግ አለባቸው? (ብዙ ምላሾች ሊኖሩ ይችላሉ) | - 1. እርግዝና ማቀድ - 2. ስለ ጤናማ እርግዝና ምክርን ለመፈለግ የጤና ተቋማትን መጎብኘት - 3. ስር ለሰደደ የጤና ችግር ምርመራ ማድረግ እና መታከም - 4. ኤች.አይ.ቪ እና የአባላዘር በሽታዎችን ምርመራ ማድረግ እና መታከም - 5. መሃንነትን መመርመር እና መታከም - 6. ለቴታነስ ክትባት መዉሰድ - 7. ልጆችን አራርቆ ለመዉለድ የቤተሰብ ዕቅድን መጠቀም - 8. ፎሊክ አሲድ መውሰድ - 9. የሰዉነት ክብደትን ማሰተካከል - 10. አመጋገብ ማሰተካከል - 11. ሲጋራ ማጨስ እና አልኮል መጠጣትን ማቆም - 12. ፅንስን የሚጎዱ እና ሕገ-ወጥ መድኃኒቶችን አለመጠቀም - 13. በስራ ፣ በከባቢ እና በሕክምና ተቋማት ውስጥ ለአላስፈላጊ ኬሚካል / የጨረር ከመጋለጥ መቆጠብ - 14. አስፈላጊ ሆኖ ሲገኝ ከዘረ-መል ጋር ለተያያዙ ችግሮች የዘረ-መል ምክር ፣ ምርመራ እና ህክምና ማግኘት - 15. ፆታዊ ጥቃት ሰለባ ለሆኑት የጤና እንክብካቤ አገልግሎቶች ፣ ሪፈራል እና የስነልቦና ድጋፍ መስጠት - 16. ከዚህ በፊት ስር የሰደደ የጤና ችግር ላለባት ሴት ከመፀነስ በፊት ምክር ፣ ምርመራ እና ህክምና ማግኘት - 17. ከዚህ በፊት ለነበረ ከእርግዝና ጋር የተያያዘ ችግር/ከህጻን ጋር የተያያዘ እክል ሕክምና ስለማድረግ ምክር ማግኘት - 18. ሌሎች (ይግለጹ) …………… |  |  |
|  | የትኛው ሥር የሰደደ የጤና ሁኔታ ፅንሱን ሊጎዳ ይችላል? (ብዙ ምላሾች ሊኖሩ ይችላሉ) | - 1. የስኳር ህመም - 2. ሥር የሰደደ የደም ግፊት - 3. ሥር የሰደደ የኩላሊት በሽታ - 4. አስም - 5. የልብና የደም ቧንቧ በሽታ - 6. ኤች.አይ.ቪ እና የአባላዘር በሽታዎች - 7. በዘር የሚተላለፍ በሽታ - 8. የሚጥል በሽታ - 9. ጭንቀት - 10. አላውቅም |  |  |
|  | የትኛውን የሕይወት ዘይቤ ወይም ባህሪ ወይም አካባቢያዊ ሁኔታ ፅንሱን ሊጎዳ ይችላል? (ብዙ ምላሾች ሊኖሩ ይችላሉ) | - 1. ሲጋራ ማጨስ - 2. አልኮል መጠጣት - 3. ሕገወጥ እጾችን መውሰድ - 4. ከመጠን በላይ ክብደት / ከትክክለኛ ክብደት በታች መሆን - 5. ለጨረር ወይም ኬሚካሎች መጋለጥ - 6. ለፆታዊ ጥቃት ሰለባ መሆን - 7. አላውቅም |  |  |
|  | ቅድመ ፅንስ እንክብካቤ እና ማማከር ለማን ያስፈልጋል ብለው ያምናሉ? | - 1. ለታዳጊዎች - 2. የመዉለጃ ዕድሜ ክልል ዉስጥ ላሉ ሴቶች - 3. ሥር የሰደደ የጤና ችግር ላለባቸው ሴቶች - 4. ቀደም ሲል የእርግዝና ችግር/ከህጻን ጋር የተያያዘ እክል ላለባቸው ሴቶች - 5. ላላገቡ ጥንዶች - 6. ለባለትዳሮች - 7. ለሁሉም ሴቶች - 8. አላውቅም |  |  |
|  | ቅድመ ፅንስ እንክብካቤ አገልግሎቶች ለምን እንደሚጠቅሙ ያውቃሉ? | - 1. አዎ - 2. አላውቅም | መልሶ አላውቅም ከሆነ ወደ ጥ.ቁ. 53 ይሂዱ |  |
|  | መልሶ አዎ ከሆነ ፣ ጥቅሞቹ ምንድ ናቸው? | - 1. የእናቶችን ጤና ማሻሻል - 2. ከሚወለዱ ህጻናት ጋር የተያያዘ እክል ለማሻሻል - 3. ያልታቀደ እርግዝና ለመቀነስ - 4. አላውቅም |  |  |
|  | ቅድመ ፅንስ እንክብካቤ ለማን ይጠቅማል? | - 1. ለህፃን ፣ ብቻ - 2. ለእናት ፣ ብቻ - 3. ለህፃን እና ለእናት - 4. አላውቅም |  |  |
|  | ሴቶች / ባለትዳሮች የቅድመ ፅንስ እንክብካቤ መቼ ማግኘት አለባቸው? | - 1. ለማርገዝ እቅድ ሲያወጡ - 2. ከመፀነስ 3 ወር በፊት - 3. በእርግዝና የመጀመሪያዎቹ 3 ወራት - 4. በሁለት እርግዝናዎች መካከል - 5. አላውቅም |  |  |
|  | ቅድመ ፅንስ እንክብካቤ አገልግሎት ለመስጠት ተገቢው ሰው ማነው? | - 1. ዶክተር - 2. የጤና መኮንን - 3. ሚድዋይፍ - 4. የጤና ኤክስቴንሽን ሠራተኛ - 5. ሁሉም ተገቢ ናቸው - 6. አላውቅም |  |  |
|  | ቅድመ ፅንስ እንክብካቤ አገልግሎቶች የት እንደሚሰጡ ያውቃሉ? | - 1. የመንግሥት ሆስፒታሎች - 2. የጤና ጣቢያዎች - 3. የጤና ኬላዎች - 4. የግል ክሊኒዎች - 5. ቤት - 6. አላውቅም |  |  |
|  | ቅድመ ፅንስ እንክብካቤ አገልግሎቶች ምን ያህል ጊዜ መሰጠት አለባቸው? | - 1. አንዳንድ ጊዜ - 2. ብዙውን ጊዜ - 3. ያለማቋረጥ - 4. አላውቅም |  |  |
|  | ቅድመ ፅንስ እንክብካቤ አገልግሎቶች በአጠቃላይ መቼ እንደሚሰጡ ሰጡ ያውቃሉ? | - 1. ከእርግዝና በፊት ወዲያውኑ በዛ ጊዜ - 2. በእርግዝና የመጀመሪያዎቹ 2 ወሮች - 3. በወሊድ ጊዜ - 4. ከወሊድ በኋላ ወዲያውኑ - 5. በሁለት እርግዝናዎች መካከል - 6. ሴቶቹ / ጥንዶች ለመፀነስ ዝግጁ በሚሆኑበት ጊዜ - 7. በማንኛውም ጊዜ ባለትዳሮች ለመፀነስ ሲያቅዱ - 8. አላውቅም |  |  |

**ክፍል 5፡ በቅድመ ፅንስ እንክብካቤ ላይ የእናቶች አመለካከት የተመለከቱ ጥያቄዎች**

| ተ.ቁ | \| **ጥያቄ** \| \| --- \| | ምላሾች | | | | |
| --- | --- | --- | --- | --- | --- | --- | --- |
|  |  | በእስ | እስ | አየ | አል | በአል |
|  | ቅድመ ፅንስ እንክብካቤ አገልግሎት እርግዝና ለሚያቅዱ ሴቶች / ባለትዳሮች ሁሉ ከፍ ያለ ቅድሚያ የሚሰጠው ጉዳይ ነው ፡፡ |  |  |  |  |  |
|  | በሕክምና የተረጋገጠ በሽታ ያለባቸው ሴቶች ብቻ ቅድመ ፅንስ እንክብካቤ አገልግሎቶችን መቀበል አለባቸው፡፡ |  |  |  |  |  |
|  | ከዚህ ቀደም ከእርግዝና ጋር የተያያዘ ችግር ያለባቸዉ ወይም ከህጻን ጋር የተያያዘ እክል ያሏቸው ሴቶች ብቻ የቅድመ ወሊድ እንክብካቤ አገልግሎቶችን መቀበል አለባቸው፡፡ |  |  |  |  |  |
|  | ቅድመ ፅንስ እንክብካቤ አገልግሎቶች የመውለድ ዕድሜ ክልል ዉስጥ ላሉ ሴቶች ብቻ መሰጠት አለበት፡፡ |  |  |  |  |  |
|  | ቅድመ ፅንስ እንክብካቤ አገልግሎቶች ላገቡ ሴቶች ብቻ መሰጠት አለበት፡፡ |  |  |  |  |  |
|  | ቅድመ ፅንስ እንክብካቤ አገልግሎቶች ላላገቡ ሴቶች ብቻ መሰጠት አለበት፡፡ |  |  |  |  |  |
|  | ቅድመ ፅንስ እንክብካቤ አገልግሎቶች ለአቅመ-ሄዋን ለደረሱ ሴቶች አስፈላጊ አይደሉም፡፡ |  |  |  |  |  |
|  | ባሎች ማንኛውንም ቅድመ ፅንስ እንክብካቤ አገልግሎት ጊዜ ከሚስቶቻቸው ጋር መሄድ አለባቸው ፡፡ |  |  |  |  |  |
|  | ቅድመ ፅንስ እንክብካቤ አገልግሎቶች በወንድ የጤና ባለሙያዎች መሰጠት አለባቸው ፡፡ |  |  |  |  |  |
|  | ቅድመ ፅንስ እንክብካቤ አገልግሎቶች በሴት የጤና ባለሙያዎች መሰጠት አለባቸው ፡፡ |  |  |  |  |  |
|  | ቅድመ ፅንስ እንክብካቤ አገልግሎቶች በልምድ አዋላጆች መሰጠት አለባቸው፡፡ |  |  |  |  |  |
|  | ቅድመ ፅንስ እንክብካቤ አገልግሎት በሚወለዱ ህፃናት ላይ ምንም ለውጥ አያመጣም ፡፡ |  |  |  |  |  |

**ክፍል 5፡ በቅድመ ፅንስ እንክብካቤ አገልግሎት አጠቃቀምን የተመለከቱ ጥያቄዎች**

| **ተ.ቁ** | \| **ጥያቄ** \| \| --- \| | **ምላሾች** | **ዝለል** | **ኮድ** |
| --- | --- | --- | --- | --- | --- |
|  | ከዚህ በፊት በነበረው እርግዝናዎ፣ ቅድመ ፅንስ እንክብካቤ አገልግሎት ከጤና ተቋም አግኝተው ያውቃሉ? | - 1. አዎ - 2. አላውቅም | መልሶ አላውቅም ከሆነ ወደ ጥ.ቁ. 73 ይሂዱ |  |
|  | መልሶ አዎ ከሆነ ፣ የትኛውን የቅድመ ፅንስ እንክብካቤ አገልግሎት ነዉ ያገኙት? | - 1. ስር የሰደደ የጤና ችግር ምርመራ እና ህክምና - 2. የአባላዘር በሽታዎችን ምርመራ እና ህክምና - 3. ኤች.አይ.ቪ ኤድስ የምክር አግልግሎት፣ ምርመራ እና ቫይረሱ በደም ዉስጥ ካለ ፀረ-ኤች.አይ.ቪ መዳኒት ማሰጀመር - 4. መሃንነትን ምርመራ እና ህክምና - 5. ለቴታነስ ክትባት መዉሰድ - 6. የቤተሰብ ዕቅድን ተጠቅሞ ልጆችን አራርቆ መዉለድ - 7. ፎሊክ አሲድ መውሰድ - 8. ከዚህ በፊት ለነበረ ስር የሰደደ የጤና ችግር ከመፀነስ በፊት ምክር ፣ ምርመራ እና ህክምና ማግኘት - 9. ከዚህ በፊት ለነበረ ከእርግዝና ጋር የተያያዘ ችግር/ከህጻን ጋር የተያያዘ እክል ክትትል እና ሕክምና ማድረግ - 10. የሰዉነት ክብደትን ማሰተካከል - 11. አመጋገብ ማሰተካከል - 12. ሲጋራ ማጨስ እና አልኮል መጠጣትን ማቆም - 13. ፅንስን የሚጎዱ እና ሕገ-ወጥ መድኃኒቶችን አለመጠቀም - 14. በስራ ፣ በከባቢ እና በሕክምና ተቋማት ውስጥ ለአላስፈላጊ ኬሚካል / የጨረር ከመጋለጥ መቆጠብ |  |  |
|  | በአሁኑ እርግዝና ወቅት የቅድመ ፅንስ እንክብካቤ አገልግሎት ከጤና ተቋም አግኝተዋል? | - 1. አዎ - 2. አላገኘሁም | መልሶ አላውቅም ከሆነ ቃለ-መጠየቁ ስላለቀ አሰናብታት |  |
|  | መልሶ አዎ ከሆነ ፣ የትኛውን የቅድመ ፅንስ እንክብካቤ አገልግሎት ነዉ ያገኙት? | - 1. ስር የሰደደ የጤና ችግር ምርመራ እና ህክምና - 2. የአባላዘር በሽታዎችን ምርመራ እና ህክምና - 3. ኤች.አይ.ቪ ኤድስ የምክር አግልግሎት፣ ምርመራ እና ቫይረሱ በደም ዉስጥ ካለ ፀረ-ኤች.አይ.ቪ መዳኒት ማሰጀመር - 4. መሃንነትን ምርመራ እና ህክምና - 5. ለቴታነስ ክትባት መዉሰድ - 6. ፎሊክ አሲድ መውሰድ - 7. ከዚህ በፊት ለነበረ ስር የሰደደ የጤና ችግር ከመፀነስ በፊት ምክር ፣ ምርመራ እና ህክምና ማግኘት - 8. ከዚህ በፊት ለነበረ ከእርግዝና ጋር የተያያዘ ችግር/ከህጻን ጋር የተያያዘ እክል ክትትል እና ሕክምና ማድረግ - 9. የሰዉነት ክብደትን ማሰተካከል - 10. አመጋገብ ማሰተካከል - 11. ሲጋራ ማጨስ እና አልኮል መጠጣትን ማቆም - 12. ፅንስን የሚጎዱ እና ሕገ-ወጥ መድኃኒቶችን አለመጠቀም - 13. በስራ ፣ በከባቢ እና በሕክምና ተቋማት ውስጥ ለአላስፈላጊ ኬሚካል / የጨረር ከመጋለጥ መቆጠብ |  |  |
|  | መልሶ አዎ ከሆነ ፣ መቼ ነዉ የቅድመ ፅንስ እንክብካቤ አገልግሎት ያገኙት? | - 1. ለማርገዝ ባቀድኩ ጊዜ - 2. እርጉዝ ከመሆኔ ከ 3 ወር በፊት - 3. በእርግዝናዬ የመጀመሪያዎቹ 3 ወራት - 4. በሁለት እርግዝናዎቼ መካከል - 5. ሌሎች (ይግለጹ) ………… |  |  |
|  | መልሶ አዎ ከሆነ ፣የቅድመ ፅንስ እንክብካቤ አገልግሎት በየትኛው ክፍል ነዉ ያገኙት? | - 1. የቤተሰብ እቅድ ክፍል - 2. ድህረ-ወሊድ አገልግሎት - 3. የማህፀን ሕክምና ክፍል - 4. ሥር የሰደደ በሽታ ክትትል ክሊኒክ - 4. ሌሎች (ይግለጹ) ………… |  |  |
|  | መልሶ አዎ ከሆነ ፣ የት ነዉ የቅድመ ፅንስ እንክብካቤ አገልግሎት ያገኙት? | - 1. የመንግሥት ሆስፒታሎች - 2. የጤና ጣቢያዎች - 3. የጤና ኬላዎች - 4. የግል ክሊኒዎች - 5. ሌሎች (ይግለጹ) ………… |  |  |
|  | መልሶ አዎ ከሆነ ፣ ማን ነዉ የቅድመ ፅንስ እንክብካቤ አገልግሎት የሰጦት? | - 1. ዶክተር - 2. ነርስ - 3. ሚድዋይፍ - 4. የጤና መኮንን - 5. የጤና ኤክስቴንሽን ሠራተኛ - 6. ሌሎች (ይግለጹ) ………… |  |  |
|  | የእርግዝና ዕቅድ ማውጣት እና በጤና እንክብካቤ አገልግሎቶች በማግኘት ላይ ውሳኔ የሚወስነው ማነው? | - 1. እኔ ብቻ - 2. ባለቤቴ ብቻ - 3. እኔ እና ባለቤቴ - 4. ሌሎች (ይግለጹ) ………… |  |  |
|  | መልሶ አዎ ከሆነ ፣ የቅድመ ፅንስ እንክብካቤ አገልግሎት በምታገኚበት ጊዜ ባልዎ ድጋፍ አድርገዉሎት እና አብሮዎት መጦ ነበር? | - 1. አዎ - 2. አይደለም |  |  |
|  | መልሶ አዎ ከሆነ ፣የቅድመ ፅንስ እንክብካቤ አገልግሎት ምን ያህል ጊዜ ያገኙት? | -------------------------------------------- |  |  |
|  | በአሁኑ እርግዝና ወቅት የቅድመ ፅንስ እንክብካቤ አገልግሎቶች አንዱ የሆነዉን ሥር የሰደደ የህክምና ሁኔታ ከተመረመሩ ፣ የትኛውን ሁኔታ ነው የተመረመሩት? | - 1. የደም ግፊት - 2. የስኳር በሽታ - 3. የሳንባ ነቀርሳ - 4. የደም ማነስ - 5. የሚጥል በሽታ - 6. መሃንነት - 7. አስም - 8. ሌሎች (ይግለጹ) ………… |  |  |
|  | በአሁኑ እርግዝና ወቅት የቅድመ ፅንስ እንክብካቤ አንዱ የሆነዉን ክብደትን ማሰተካከል ከተገበሩ፣ ምን ዓይነት የክብደት ማሰተካከል ነዉ የተገበሩት? | - 1. ጤናማ ክብደት መጠን ላይ መድረስ - 2. ክብደት መቀነስ |  |  |
|  | በአሁኑ እርግዝና ወቅት የቅድመ ፅንስ እንክብካቤ አገልግሎት አንዱ የሆነዉ ፎሊክ አሲድ ከወሰዱ ፣ በቋሚነት ሲጠቀሙ ነበር? | - 1. አዎ - 2. አልነበረም |  |  |
|  | በአሁኑ እርግዝና ወቅት የቅድመ ፅንስ እንክብካቤ አንዱ የሆነዉን አመጋገብን ካስተካከሉ፣ ምን አይነት የአመጋገብ ማስተካከያ ነዉ የወሰዱት? | - 1. ከፍተኛ ካርቦሃይድሬት መጠን ያላቸውን ምግቦች መተዉ - 2. ከፍተኛ ቅባት/ስብ መጠን ያላቸው ምግቦች መተዉ - 3. ከፍተኛ ስኳር መጠን ያላቸውን ምግቦች መተዉ - 4. የተመጣጠነ ከተለያዩ የምግብ ዓይነቶች ቶሎ ቶሎ መመገብ - 5. ሌሎች (ይግለጹ) ………… |  |  |

**ስለ ትብብሮ አመሰግናለሁ**
