# Supplementary material for: Utilization of preconception care and associated factors in Hosanna Town, Southern Ethiopia
Source: PLoS One. 2022 Jan 7;17(1):e0261895. doi: 10.1371/journal.pone.0261895 (PMC8741054; doi:10.1371/journal.pone.0261895)
Supplement: Attachment — Submitted filename: Utilization of preconception care and associated factors in Hosanna Town, Southern Ethiopia.docx [file pone.0261895.s004.docx]

Comments to the Authors

This paper aims use a cross-sectional to investigate the Utilization of preconception care and associated factors in Hosanna Town, Southern Ethiopia. It’s interesting, however, my overall impression of this work is that it is not ready for publication owing to the following reasons:

This article is quite an interesting research, but could not clearly see each Questionnaire's relevant references in the introduction section. for example, Measurement section, p136~167, for more contribution, it’s would be better-cited references. In addition, suggest authors statistic analysis by professional proof.

- Abstract Result section, please check related utilization of preconception care data, page 41~46, page 284~289, and Page 332~334 table 4, because the authors’ table presents that COR(95% CI) is incorrect.
- Page 196~198 : “Three hundred twenty seven 197 (81.8%) were Hadiya….” And “More than half of women were housewives 257 (64.3%). Nearly…”Please check, I could not find them in table 1.
- Page 204~206: Reproductive characteristics section, “Nearly half of 206 the women had never given birth 186 (46.5%). The median age at first birth was 21 (IQR 19, 24). 207 Out of women who had 2 or more children, about one third had birth…” and “More than half 209 of the women had received antenatal care two to three times 227 (56.8%)….”Please check, I could not find those data in table 2.
- Page 210~212: please serious care your data, “More than one quarter of the women had ever used any family planning method 109 (27.3%). More than one fourth of the women had used modern family planning before current pregnancy 116 (29.0%).” Please check, I could not got them in table 2.
- Page 234~236, please check table 2, where authors miss some data, such usthe total samples 400, but Parity total number only 214, Birth interval total number only 90, Type of family planning used total number only 116, Planned any of previous pregnancies total number only 234, please carefully deal with them.
- table 3 and table 4, all data had the same problem with table2, please carefully deal with them. So authors should be rewording Obstetrics and medical characteristics section base on incorrect table 3.
- Page 225~230: Health service-related factors section, authors should rewording, because figure 4 could not find “Majority of women resided within 5kms of health facility 379 (94.8%)and almost all women had 227 no challenge in accessing health facility 394 (98.5%). Thirty-four (8.5%) women had received counseling on preconception care in previous pregnancies…”  please carefully deal with data.
- P242~267, Knowledge on preconception care section, base on the study’s total samples 400, why some missing data not present in table,  pleases rewording the part base on your data.
- P268~278, Attitude towards preconception care section, the same question with the incorrect tables.
- P279~302, Preconception care utilization section, the same question with the incorrect tables.
- P303~324, Factor sassociated with preconception care section, the same question with theincorrect tables.
- P337~428, Discussion section, the same question with the incorrect tables.
- The limitation part, so few, it’s should add more related the study.
- In the discussion section, suggest you add your finding linked with the study’s

hypothesis.

- Some references incorrect, authors should follow PLoS one guidelines style.
- Thank you for your efforts.
