# Supplementary material for: Utilization of preconception care and associated factors in Hosanna Town, Southern Ethiopia
Source: PLoS One. 2022 Jan 7;17(1):e0261895. doi: 10.1371/journal.pone.0261895 (PMC8741054; doi:10.1371/journal.pone.0261895)
Supplement: Attachment — Submitted filename: Rebuttal Letter.docx [file pone.0261895.s005.docx]

**Rebuttal Letter**

We would like to express our heartfelt appreciation for offering us chance to re-send the manuscript after outlining our responses for the reviewer’s comments. Having said this we have presented our responses for the comments forwarded as follows:

Academic editors

Comment: Please ensure that your manuscript meets PLOS ONE's style requirements, including those for file naming.

Response: We have developed the manuscript based on PLOS ONE’S formatting style.

Comment: Please include your actual numerical p-values in Table 4.

Response: We have included the actual p-value in Table 4.

Comment: In your methods, please provide the names of the four public health institutions in your study.

Response: We have included the names of the four public health institutions.

Comment: PLOS requires an ORCID ID for the corresponding author

Response: We have included the ORCID ID for the corresponding author.

Reviewer #1:

Comment: This article is quite an interesting research, but could not clearly see each Questionnaire's relevant references in the introduction section. For example, Measurement section, p136~167, for more contribution, it’s would be better-cited references. In addition, suggest author’s statistical analysis by professional proof.

Response: We have cited reference for the measurements described from line 136-167. We do believe that statistical analysis were performed appropriately. The evidence is that the goodness of fit of the model was checked using Hosmer and Lemeshow test of assumption. It had a chi-square value of 8.067 & p-value of 0.327 which showed the model to be fit.

Comment: Abstract Result section, please check related utilization of preconception care data, page 41~46, page 284~289, and Page 332~334 table 4, because the authors’ table presents that COR (95% CI) is incorrect.

Response: Line 41-46 is describing the utilization of preconception care & we have put the Confidence interval this utilization at 95% Cl. Line 284-289 is describing about different issues regarding preconception care among those who have utilized preconception care. The COR presented in Table 4 is correct & it can be checked manually, i.e. OR=ad/bc.

Comment: Page 196~198: “Three hundred twenty seven 197 (81.8%) were Hadiya….” And “More than half of women were housewives 257 (64.3%). Nearly…”Please check, I could not find them in table 1.

Response: We have inserted these figures in to the table 1.

Comment: Page 204~206: Reproductive characteristics section, “Nearly half of 206 the women had never given birth 186 (46.5%). The median age at first birth was 21 (IQR 19, 24). 207 Out of women who had 2 or more children, about one third had birth…” and “More than half 209 of the women had received antenatal care two to three times 227 (56.8%)….”Please check, I could not find those data in table 2.

Response: We have inserted data regarding women who had given birth. However, data on median age at first birth is not included in the table since median is not categorical variable & therefore we have simply stated it in words. The statements saying “Out of women who had 2 or more children” were already described in the table (check on birth interval). But we have inserted frequency of antenatal care in to the table.

Comment: Page 210~212: please serious care your data, “More than one quarter of the women had ever used any family planning method 109 (27.3%). More than one fourth of the women had used modern family planning before current pregnancy 116 (29.0%).” Please check, I could not got them in table 2.

Response: We have inserted data regarding ever used any family planning method. For the statements saying “More than one fourth of the women had used modern family planning before current pregnancy” we have changed before current pregnancy into “recently” to avoid confusion.

Comment: Page 234~236, please check table 2, where authors miss some data, such us the total samples 400, but Parity total number only 214, Birth interval total number only 90, Type of family planning used total number only 116, Planned any of previous pregnancies total number only 234, please carefully deal with them.

Response: In the case of parity; the total number is 214 due to the fact that in the current study, 186 women were primigravida (getting pregnant for the first time). Similarly, to estimate the birth interval; the study participants need to have at least 2 children & therefore there were 90 women having children 2 & more. The type of family planning is used was only 116 due to the fact that these were the women using family planning methods & so from those using family planning (1116), various FP methods were described in the table. The statement saying “planned any of previous pregnancies….” is from those who have at least got pregnant once previously & therefore the total was 234.

Comment: table 3 and table 4, all data had the same problem with table2, please carefully deal with them. So authors should be rewording Obstetrics and medical characteristics section base on incorrect table 3.
Response: All the statements written under obstetrics & medical characteristics are found in table 3. We believe that the text & table here have no problem. We kindly request the reviewer to check this in table 3. Concerning table 4; we have referred it in text & we have refined it.

Comment: Page 225~230: Health service-related factors section, authors should rewording, because figure 4 could not find “Majority of women resided within 5kms of health facility 379 (94.8%) and almost all women had 227 no challenge in accessing health facility 394 (98.5%). Thirty-four (8.5%) women had received counseling on preconception care in previous pregnancies…” please carefully deal with data.

Response: The statements stating “Majority of women resided within 5kms of health facility 379 (94.8%) and almost all women had no challenge in accessing health facility 394 (98.5%) has no figure. But the figure is for the statement “Thirty four (8.5%) women had received counseling on preconception care in previous pregnancies and commonly received counseling were diet modification before conception 14 (41.2%) and counseling on folic acid supplementation 6 (17.6%).” We have now separated the two statements.

Comment: P242~267, Knowledge on preconception care section, based on the study’s total samples 400, why some missing data not present in table, pleases rewording the part base on your data.

Response: We have described knowledge on preconception care only by text. We don’t have tables for this section. We don’t have missing for knowledge on preconception section due to the fact that every participant’s level of knowledge have been assessed. Here, we have reported major findings using text. For example in the line 245 it says, “one hundred seventy (42.5%) respondents didn’t know chronic medical conditions that affect the fetus.” What we understand from this statement is; 170 women didn’t know about chronic medical condition that affect the fetus while 230 women knew & mentioned some chronic medical condition that affect the fetus. Therefore, we kindly request the reviewer to understand the statements under knowledge in such sense.

Comment: P268~278, Attitude towards preconception care section, the same question with the incorrect tables.

Response: Similarly, here also we don’t have tables for attitude section. We have described the findings only by text. We have reported the major findings using text.

Comment: P279~302, Preconception care utilization section, the same question with the incorrect tables.

Response: Here also, we don’t have tables for preconception care utilization section. What we have is figure 2. Again, we have reported the major findings using text.

Comment: P303~324, Factors associated with preconception care section, the same question with the incorrect tables.

Response: We have referred table 4 in text & added table 4 in the first two paragraphs under this section.

Comment: -P337~428, Discussion section, the same question with the incorrect tables

Response: the discussion section has no tables. The discussion part is presented using text.

Comment: -The limitation part, so few, it’s should add more related the study.

Response: We have added additional points on the limitation part.

Comment: In the discussion section, suggest you add your finding linked with the study’s hypothesis.

Response: We have discussed based on the objectives of the study. The two objectives were to assess preconception care utilization and associated factors among pregnant women attending antenatal care clinics of public health facilities in Hosanna town. Based on this two objectives, we have presented the discussion section. We kindly request the review to consider it in such manner.

Comment: Some references incorrect, authors should follow PLoS one guidelines style.

Response: We have corrected the reference as per comment given.

Reviewer #2:

Comment: In the introduction, it should be emphasized better why it is important of preconception care utilization for the region where you work.

Response: We have corrected the introduction part as per comment given.
