# Supplementary material for: Utilization of preconception care and associated factors in Hosanna Town, Southern Ethiopia
Source: PLoS One. 2022 Jan 7;17(1):e0261895. doi: 10.1371/journal.pone.0261895 (PMC8741054; doi:10.1371/journal.pone.0261895)
Supplement: Attachment — Submitted filename: Rebuttal Letter.docx [file pone.0261895.s006.docx]

**Rebuttal Letter**

We would like to express our heartfelt appreciation for offering us chance to re-send the manuscript after outlining our responses for the reviewer’s comments. Having said this we have presented our responses for the comments forwarded as follows:

Reviewer #1:

Comment: Questionnaire's relevant references are not presented from lines 136-167.

Response: We have cited reference for the measurements described from line 136-167.

Comment: In addition, the author’s statistical analysis stated that it performed appropriately. But table 2, table 3, and table 4 still reported incorrect data; Statistical information is incorrect, easy to mislead readers.

Response: We have corrected table 2 & table 3 as per comment given. However, we believe that table 4 has no incorrect data. The variable “previous history of adverse birth outcome” has missing data due to the fact that primigravida women will not have history of adverse birth outcome as they have not been pregnant previously. We handled this using advanced statistics (multiple imputation). We kindly request the reviewer to check this in table 4.

Thank you for your constructive comments.
